# Uncovering Disparities in Medical Education: Advisory Guide of Strategies and Best Practices for Illuminating Blind Spots

**DOI:** 10.1007/s40670-025-02435-3

**Published:** 2025-08-15

**Authors:** Laurah Turner, Lauren J. Germain, Jeffrey Bird, Michael S. Ryan, Marjorie Westervelt, Josie Suser, Sally A. Santen

**Affiliations:** 1 University of Cincinnati College of Medicine, Cincinnati, USA; 2 SUNY Upstate Medical University, Syracuse, USA; 3 Zucker School of Medicine at Hofstra/Northwell, Hempstead, USA; 4 University of Virginia School of Medicine, Charlottesville, USA; 5 Baylor College of Medicine, Houston, USA; 6 Dept. of Emergency Medicine, University of Cincinnati College of Medicine, 231 Albert Sabin Way, ML 0769, Cincinnati, OH 45267-0769 USA; 7 Virginia Commonwealth University, Richmond, USA

**Keywords:** Assessment, Grading, Undergraduate medical education, Fairness

## Abstract

Assessment and grading are prone to disparities based on race and ethnicity. Health professions educators need a guide for how to strategically navigate assessment data. This monograph provides a consensus-driven advisory guide, drawing upon common challenges schools face when detecting, measuring, and analyzing gender/racial disparities, and shares practical algorithms and strategies for engaging with the data. Five steps are proposed: create a process diagram; identify assessment data sources and potential manifestations of disparities and inequities in assessments; consider other variables when analyzing data; choose analytical approaches to evaluate sources of disparities; and identify potential threats and challenges.

## Introduction

Assessment and grading in undergraduate medical education are prone to disparities due to race and ethnicity [1–7]. Disparity refers to outcomes that differ between groups [8, 9]. These disparities may originate from various sources, including rater bias (implicit or explicit) and measurement bias [5]. Despite calls for action from US medical schools and foundations [6–9], few resources exist detailing how institutions can determine whether such disparities exist and indicate potential problems. Moving to identify disparities and address potential biases will create more equitable learning experiences and improve medical education [5].

Disparity, inequity, and bias are distinct but related concepts. While disparity describes differences between groups, inequity refers to a lack of fairness in the treatment of individuals or groups [8, 9]. Bias refers to a preference towards or against a particular group or individual, often based on stereotypes or preconceptions [8, 9]. Disparity alone is not evidence of inequity or bias. For example, we may observe that students who matched into general surgery had higher grades in their surgical clerkship than those who matched into pediatrics. This is evidence of disparity but is not sufficient to demonstrate inequities or biases in grade assignment. When we observe disparities between groups, it requires us to interrogate the system to identify potential sources of inequity or bias [10] to be able to intervene [11]. In medical education, similar logic applies—disparities in assessment outcomes (for example, differences in clinical grades between groups) may signal implicit bias or systemic inequities within the educational system. For instance, Low et al. and others document how measurable differences in clinical grading among medical students can reflect these underlying issues [1, 12–14].

Medical schools need a guide for how to strategically navigate assessment data, identifying disparities, and beginning to define and address potential biases to create a fair assessment system leading to more equitable learning experiences and improve medical education. There are published papers addressing aspects of grading disparities [2–7]. Teherani et al. note how small differences in assessed clinical performance amplify to create meaningful differences in grades and awards [4]. The Macy conference proceedings review aspects of bias in assessment across learner groups and make recommendations to address bias [7]. This monograph provides an advisory guide of a stepwise approach to explore disparities in medical school assessment data.

Briefly, our methods were to bring together the literature and the expertise and experience of a data-focused subgroup of Medical Educators Dedicated to Collaboration for the Assessment, Representation & Equity (MEDCARE) community of practice. This is a group of six schools across the USA that created a group with shared aims to identify and address grading disparities. Our group of schools has spent the past 3 years sharing our approaches to exploring grading disparities. We started with literature on bias in assessment, and then each school completed school-based analyses of differences. The strength of the group was the different approaches for each school and sharing practices. The process of creating this advisory guide was a series of Zoom meetings. The author group iteratively discussed approaches and, through writing this monograph, developed consensus to provide the advisory guide, drawing upon the literature, common experiences, and challenges medical schools face when detecting, measuring, and analyzing gender and racial/ethnic disparities and sharing practical algorithms and strategies for engaging with the data. We have presented our approach at the Association of American Medical Colleges meeting for feedback with revisions.

## Context: Identifying a (Potential) Problem (Step 0)

Medical schools collect a massive volume of assessment data so it may not be feasible or high yield to interrogate every assessment point. Therefore, the preliminary step is determining the domain of focus—such as investigating disparities in medical school grades or comments in narrative assessments. If, for example, one observes that students from underrepresented groups received approximately half the numbers of “honors” across core clerkships [4], it is prudent to further examine clerkship assessment and grading systems.

As the objective is to identify grading disparities and bring potential inequities to light, decide which socio-demographic categories (i.e., gender, race, ethnicity) warrant examination based on the local context, identify data sources, and be explicit about definitions (and limitations to the definitions). One option is to use American Medical College Application Service admissions data, which enables students to identify themselves.

Establish data governance guidelines concerning the sample student population. For instance, some students may not remain with their matriculating cohort, with reasons for delay potentially linked to academic challenges. Consequently, data rules and definitions must be defined for consistent student inclusion and exclusion criteria within a cohort. Careful documentation is critical.

## Step 1: Create a Process Diagram

**Fig. 1:**
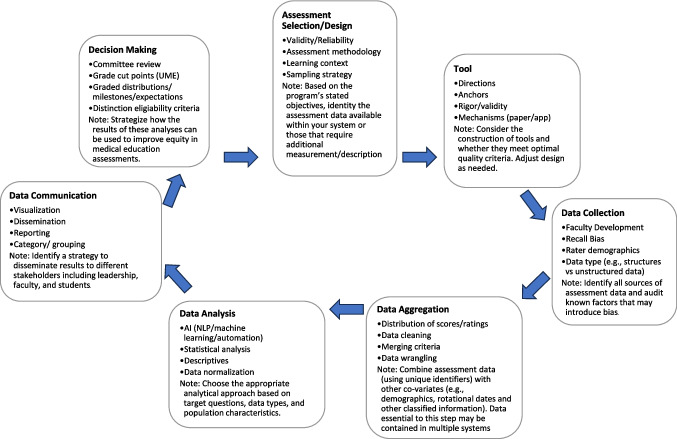
Process diagram of considerations to determine, collect, analyze, and judge assessment data

A process diagram can be used to map the complicated system of assessment as well as potential sources of biases. Start with educational objectives and end with outcomes. Consider including the following activities to determine if they are part of your assessment process: (1) assessment selection; (2) assessment tool design (including rater training); (3) data collection; (4) data aggregation; (5) data analysis; (6) data communication; and (7) decision-making (Fig. 1). Under each step of the diagram, list variables that impact the immediate outcome of the step and/or the overarching educational objectives. This list of variables will prove helpful when identifying potential sources/expressions of disparities in assessments (Steps 2 and 4).

## Step 2: Identifying Assessment Data Sources and Potential Manifestations of Disparities and Inequities in Assessments

Utilizing the process diagram from Step 1, educators can identify potential sources of disparity and underlying biases. Determine the assessments employed and their relative influence on determining an outcome (e.g., final grade). There is evidence that students from groups underrepresented in medicine (URiM) can have lower standardized exam (e.g., United States Medical Licensing Examination Step 1 and Step 2 exams) [15] and clinical evaluation scores [5] and different narrative assessments of their performance [2] than peers. All of this has been shown to impact clerkship grades [1], class rank, Alpha Omega Alpha Honor Society[16], and other high-stakes decisions [1, 4].

## Step 3: Other Variables for Consideration When Analyzing Data

A student’s outcome (i.e., grade) is driven by a combination of student factors (knowledge, skills, and attitudes), the environment in which they learn, and the system of grading itself. It is therefore essential to recognize covariates or independent variables that might influence outcomes. This might include site, role (attending or resident), assessor socio-demographic factors, timing, and learning environment characteristics. When collecting and analyzing data, institutions should be aware that certain variables present specific challenges to obtain. Protected information such as assessor demographics may be restricted by privacy policies and not readily linked to assessment data in institutional systems. Historical data about past experiences of assessors or students may be stored in separate systems or not consistently documented across time periods. Environmental factors such as team dynamics, patient volume and acuity, or institutional culture are rarely quantified systematically but may significantly impact assessment outcomes. Similarly, interpersonal variables like the quality of the student-assessor relationship or frequency of meaningful interactions are difficult to measure objectively but may substantially influence ratings. To determine which variables have more influence on potential bias, institutions should conduct preliminary analyses to identify correlations between available variables and assessment outcomes, review relevant literature on assessment bias in medical education, consider using mixed-methods approaches including qualitative feedback, and test different variable combinations to determine which explain the most variance in outcomes. These analyses will help institutions weigh these factors when deciding which metrics to collect and include in their analytic model, balancing the effort required to obtain difficult variables against their potential explanatory value for understanding disparities.

## Step 4: Analytical Approaches (Quantitative and Qualitative) to Evaluate Sources of Disparities

Upon aggregating the data and defining specific assessment questions, select the appropriate data analysis method to detect potential disparities. The choice of analytical approach depends on the nature of the data, which can be categorical (i.e., nominal or ordinal), continuous (i.e., interval or ratio), or narrative. For many educators, this entails seeking expert assistance in conducting either quantitative or qualitative analyses.

To discern disparities in quantitative data, the appropriate statistical test should be chosen based on the type of data (categorical or continuous) and the number of groups being compared (Table 1). As sample sizes vary, a power analysis can be used to determine whether one can draw conclusions from the results. The team should prospectively identify the effect size and/or statistical significance that constitutes a meaningful difference while recognizing the limitations of small samples and the potential need for multi-year analyses.

**Table 1:** Analysis options

Research question	Assessment scenario	Suitable statistical test	Example medical education assessment
Is there a significant difference in medical student scores before and after participating in an intervention?	Comparing medical student performance over time	Paired *t*-test (paired samples) (assuming normally distributed data)	Pre- and post-training scores of medical students who participated in an intervention
Is there a significant difference in performance of two groups over time?	Comparing standardized patient scores before and after	Wilcoxon signed-rank test (non-parametric alternative) (data not normally distributed)	Rating scores of medical students before and after a communication workshop
Are there differences in exam scores between two groups?	Comparing two groups	Student’s *t*-test (independent samples)	Exam scores of students who attended a traditional lecture vs. online lecture
Are the differences in performance between two groups?	Comparing two groups	Mann–Whitney *U* test (non-parametric alternative if data not normally distributed)	Comparing teamwork between URiM and non URiM
Are there differences in performance among multiple groups?	Comparing more than two groups	One-way ANOVA with post hoc tests (e.g., Tukey, Bonferroni)	Performance scores of students across multiple medical schools
Are there differences in performance among multiple groups?	Comparing more than two groups	Kruskal–Wallis test (non-parametric alternative) (data not normally distributed)	Rankings of different residency programs based on various criteria
		Chi-square test (categorical data)	Gender distribution among different medical specialties
Is there an association between study hours and exam performance?	Correlation between two variables	Pearson correlation coefficient	Association between hours of study and exam performance
		Spearman’s rank correlation coefficient (non-parametric alternative)	Relationship between student satisfaction ratings and faculty evaluations
Can GPA be used as a predictor of success in medical school?	Predicting outcomes	Simple linear regression	GPA as a predictor of success in medical school
		logistic regression	Predicting the likelihood of students passing the licensing exam
Are there differences in proportions across different levels of grading (Honors, High Pass, Pass)?	Comparing proportions	Chi-square test	Proportion of medical students earning various scores
		Fisher’s exact test (small sample sizes)	Adverse events experienced by students during clinical rotations
Is there agreement in assessing clinical skills between different raters?	Assessing agreement or reliability	Cohen’s kappa coefficient (categorical variables)	Inter-rater agreement in assessing clinical skills

When considering covariates, decide whether to include them in the analysis to unveil more nuanced differences, particularly when investigating data for potential inequities. More sophisticated analytical tests could aid in identifying potential confounding variables that influence analyses of bias and disparity.

Disparities and biases also manifest in narrative comments [2]. While quantitative analyses provide statistical evidence of disparities, qualitative approaches offer insights into the language, tone, and context of assessments. For analyzing narrative data, we recommend:

1. **Thematic analysis**: Using established methods to identify patterns within narrative comments. This process involves familiarization with data, generating initial codes, searching for themes, reviewing themes, defining themes, and reporting findings [17].
2. **Software-assisted analysis**: Tools such as NVivo, MAXQDA, or QDA Miner can facilitate coding and analysis of large datasets of narrative comments. These programs allow for systematic organization of qualitative data and identification of thematic patterns [18].
3. **Linguistic analysis**: Examining word choice, sentence structure, and tone can reveal subtle biases in assessments. For example, research has shown differences in the use of standout adjectives (“exceptional,” “outstanding”) and grindstone traits (“hardworking,” “diligent”) between demographic groups [19, 20].
4. **Inter-rater reliability**: When analyzing narrative comments, having multiple coders independently review the data enhances reliability. Calculate Cohen’s kappa or Fleiss’ kappa to assess agreement between raters [21].

The language used in the comments should be examined along with the context in which the comments were made. Employing a diverse team of analysts to review the narrative data can assist in detecting potential bias and providing a more comprehensive analysis.

When group-based disparities are identified in any dataset, the next step is a deeper analysis to determine to what extent the disparities are due to biases. It is important to engage the team and other invested parties in this step. Return to the process diagram (Step 1) to establish which variables warrant further consideration. Comparing narrative and quantitative data can be beneficial in this step, as mixed-methods approaches often provide more comprehensive insights than either method alone.

To address ethical considerations, institutions should comply with Federal Education Rights and Privacy Act regulations, leverage internal review mechanisms such as Institutional Review Boards (IRBs), and uphold ethical standards in managing and communicating findings. A small review committee may be useful at the collection, analysis, and dissemination phases of each project.

## Step 5: Identifying Potential Threats and Challenges

Lastly, acknowledging potential threats and challenges to the process is crucial. A significant concern is the small sample sizes of URiM students in many schools (less than 10 students per class) [22], coupled with the categorization of learners from diverse racial backgrounds. One option is schools may choose to aggregate multiple groups into a single category of underrepresented in medicine. However, there may be meaningful outcome differences between different racial or ethnic groups. Another option is to aggregate students across multiple years. Mostly, it is important to recognize and communicate the limitations so that broad generalizations are not made based on small sample sizes. Additional challenges encompass alterations in the curriculum and grading systems over time. Assessment data may reveal disparities and potential bias. Thus, the judgments by course/clerkship directors, or entrustment, competency, and grading committees regarding these data can also introduce bias. Ultimately, the sensitive nature of this issue requires a carefully crafted communication strategy addressing multiple groups of stakeholders [4].

## Applying the Advisory Guide in Practice

The MEDCARE community of practice schools applied this process to explore differences in assessment outcomes during the clerkship phase of medical education. Schools identified various assessment points showing disparities, including USMLE Step examinations, clerkship honors distributions, and clinical assessment components. When disparities were identified, schools engaged in careful analysis to determine potential sources of bias following the process outlined above.

While specific institutional findings are not presented here (as the purpose of this monograph is to provide methodological guidance rather than institutional case studies), the consistent finding across institutions was that the systematic process outlined in this guide helped identify areas for improvement in assessment practices. Schools reported that having a structured approach facilitated data-driven conversations about fairness that might otherwise have been difficult to initiate. The process also highlighted the importance of considering multiple variables and assessment methods when evaluating equity in medical education.

## Further Guidance

While disparities and inequities in assessment in medical education have been acknowledged, there is little guidance on how institutions might identify problematic patterns in local data. There may also be institutional blind spots where there is a hesitancy to explore data due to contextual factors. The purpose of this advisory is to provide a consensus-driven 5-step process for medical schools who wish to identify patterns of disparities in their local assessments. The guide is designed to standardize the approach to exploration and provide concrete strategies to help schools navigate this difficult process, fraught with potential landmines.

Across institutions, some forms of assessment were more prone to seeing or analyzing differences than others (e.g., written exams) [23]. Determining whether or to what the disparities were due to biases involved iterative conversation and reflection regarding assessment development, implementation, and analysis among group members. Such conversations were effective in uncovering some sources of bias because the environment of collective action was protective.

This guide is only one step along the way to identifying solutions for the long-known disparities identified in medical assessment data. It is essential to be thoughtful in communicating the results of the data exploration process to different key groups such as URM students, faculty, curriculum leaders, and deans, considering the sensitivity of the subject matter. Implementing the strategies outlined in this guide may help medical schools effectively address the disparities and biases that exist in their assessment processes, ultimately improving medical education for all students. By fostering a more equitable learning environment, medical schools can better prepare their students for successful careers as healthcare professionals, dedicated to serving diverse patient populations with the highest quality of care.
